# Management of gastric conduit dehiscence with self-expanding metal stents: a case report on salvaging the gastric conduit

**DOI:** 10.1186/s13019-017-0570-z

**Published:** 2017-01-25

**Authors:** Diana H. Liang, Leonora M. Meisenbach, Min P. Kim, Edward Y. Chan, Puja Gaur Khaitan

**Affiliations:** 1 Division of Thoracic Surgery, Department of Surgery, Houston Methodist Hospital, 6550 Fannin Street, Suite 1661, Houston, TX 77030 USA; 2 Weill Cornell Medicine, Houston Methodist Hospital, Houston, TX USA

**Keywords:** Esophageal stents, Esophageal surgery, Operations

## Abstract

**Background:**

Three-hole minimally invasive esophagectomy (3HMIE) is one of the most radical procedures in gastrointestinal surgery. It involves thoracoscopic dissection of the esophagus followed by creation of a gastric conduit in the abdomen with anastomosis in the neck, and is associated with significant morbidity. Gastric conduit dehiscence is one of the most morbid complications following esophagectomy. Historically, the standard of care in this situation has been conduit diversion with delayed esophageal reconstruction.

**Case presentation:**

Here, we report two patients with a timely diagnosis of gastric conduit dehiscence of staple line after 3HMIE who were salvaged successfully with endoscopic placement of self-expanding metal stents.

**Conclusion:**

Endoscopic stents may be used in selected cases of gastric conduit dehiscence after 3HMIE to salvage the conduit.

## Background

Three-hole minimally invasive esophagectomy (3HMIE) is used to treat patients with esophageal cancer and benign end-stage esophageal disease. Gastric conduit necrosis and dehiscence remains a rare, but catastrophic, complication after any esophagectomy [1]. Traditionally, such complications have necessitated conduit removal and esophageal diversion with esophagostomy followed by delayed reconstruction with either jejunum or colon [1]. Here, we present two patients with partial gastric conduit necrosis and/or dehiscence with thoracic contamination after 3HMIE that were successfully managed with self-expanding metal stents (SEMS) with concomitant chest decortication.

## Case presentation #1

**Fig. 1:**
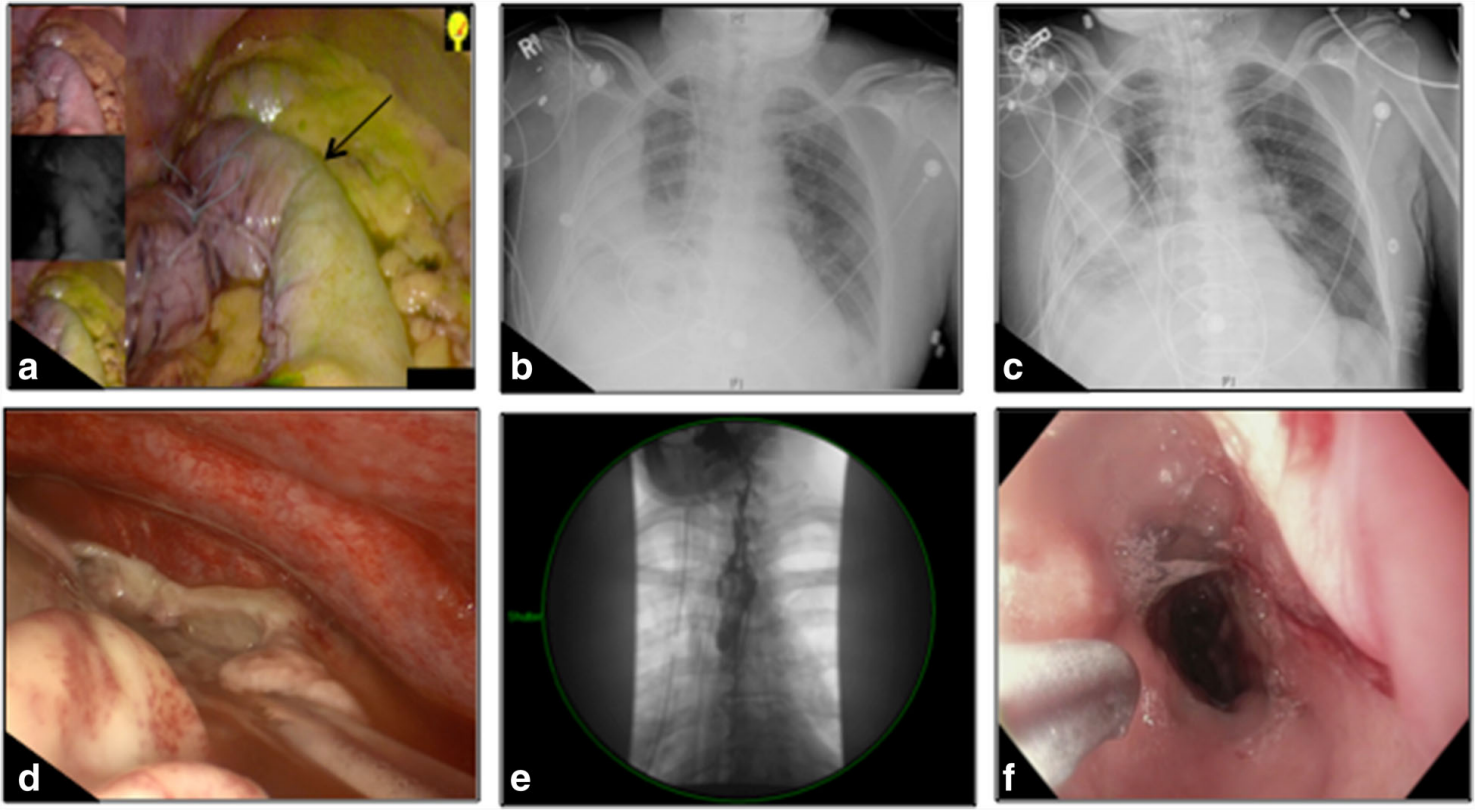
Case #1. A clear demarcation line between the well-perfused and marginally-perfused conduit was noted (*black arrow*, **a**). Postoperative chest X-ray demonstrated right-sided opacification despite an indwelling tube (**b**), which did not significantly improve with placement of an additional tube (**c**). On thoracoscopic exploration, a multiloculated effusion with significant rind was seen (**d**). A partially-covered self-expanding metal stent was placed (**e**), and the conduit healed after 2 serial stents (**f**)

A 65-year-old male who had received definitive chemoradiation therapy for cuT3N0 esophageal squamous cell carcinoma (SCC), with an initially complete clinical response, was found to have recurrent cancer 2 years later located at 30 cm from the incisors on surveillance endoscopy. He underwent a salvage 3HMIE. A gastric conduit was constructed using serial firing of staplers. After creation of the conduit, perfusion was confirmed using Pinpoint fluorescence angiography (Novadaq Technologies Inc, Ontario, Canada) (Fig. 1a). A side-to-side, but functional end-to-end, esophago-gastric anastomosis was created in the left neck using staplers below the transition point. On postoperative-day (POD) 3, the patient developed acute respiratory distress and a CXR demonstrated a partial white-out on the right (Fig. 1b). He was emergently intubated, and a bedside bronchoscopy was performed which did not demonstrate a mucus plug. Therefore, a right-sided pleural effusion was suspected and a second chest tube was placed that did not resolve the effusion (Fig. 1c). Due to a concern for an anastomotic leak, he was taken for a thoracoscopic exploration and neck drainage. Multiple serous fluid collections were found in the right chest with a significant amount of diffuse thick rind, requiring decortication (Fig. 1d). An on-table endoscopy revealed ischemic changes of the gastric conduit spanning about 2 cm at 24 cm from the incisors. The anastomosis was intact at 20 cm. This suggested partial necrosis of the conduit and leak from the staple line into the thoracic cavity. A 23 mm × 155 mm WallFlex partially-covered self-expanding metal stent (pcSEMS, Boston Scientific, Natick, MA) was deployed under fluoroscopy, and bridled in place using umbilical tape. A completion esophagram confirmed appropriate stent placement without an active leak (Fig. 1e). Final surgical pathology returned as ypT1bN0 stage IB SCC. Twenty-five days after the pcSEMS placement, repeat endoscopy and stent retrieval was performed. Upon stent removal, macerated mucosa underneath the stent was seen with a 1 cm defect approximately 4 cm below the anastomosis. An on-table esophagram confirmed a small contained leak at the gastric conduit staple line, which prompted re-placement of a pcSEMS. Two weeks later, the second pcSEMS was removed, and the leak site was found to be completely healed. He was started on a liquid diet and gradually advanced to a regular diet. Two months later, the patient developed a stricture just below the anastomosis (Fig. 1f) that required serial balloon dilations with Kenalog (Bristol-Myers Squibb, Princeton, NJ) injection with complete resolution of symptoms after 6 months.

## Case presentation #2

**Fig. 2:**
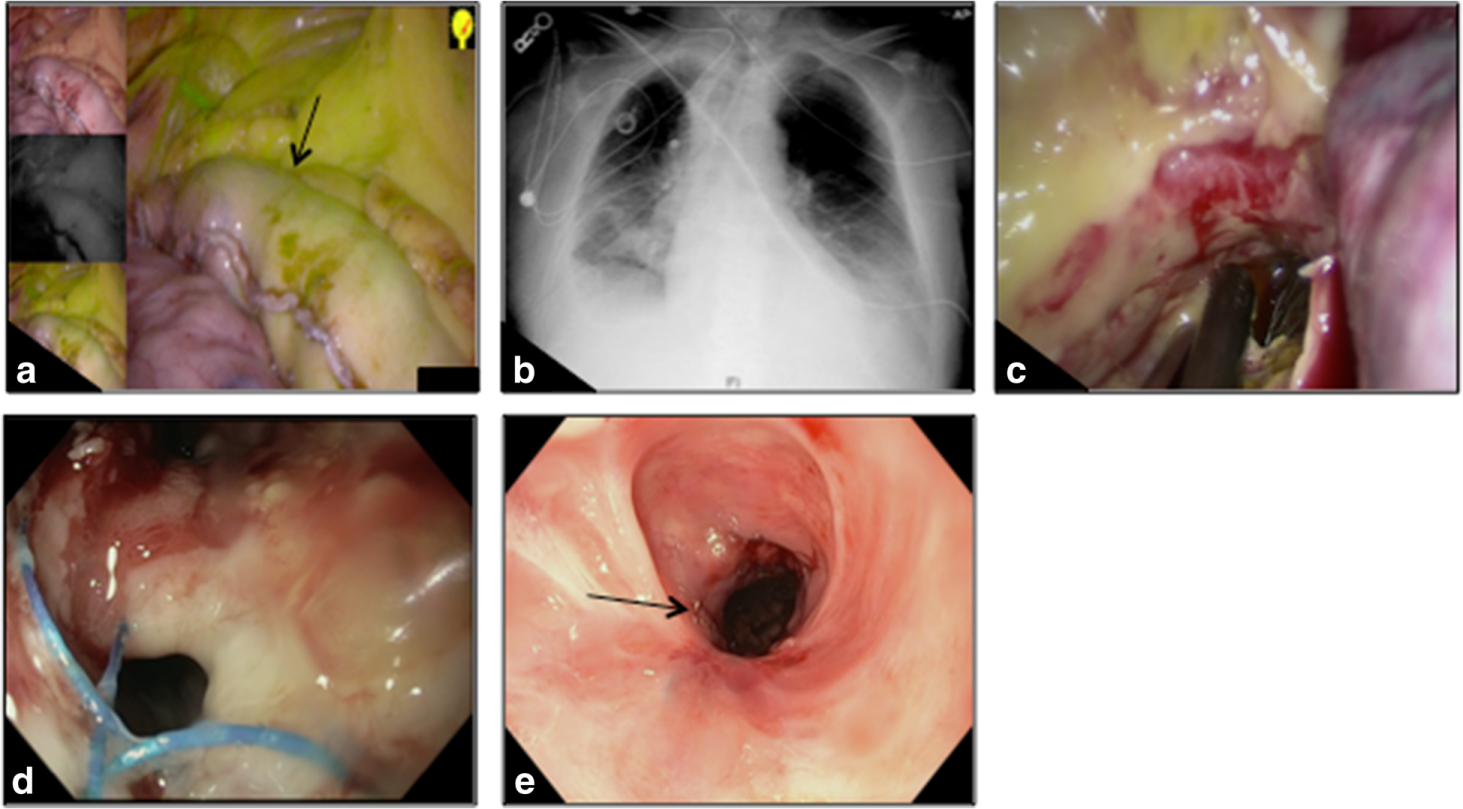
Case #2. A demarcation line between the well-perfused and marginally-perfused regions (black arrow, **a**). Once a leak was identified, a partially-covered self-expanding metal stent was placed (**b**). Partially exposed esophageal stent seen in the apex of the chest on thoracoscopic exploration (**c**; shown at the tip of the thoracoscopic suction catheter). Visible defect in conduit during repeat endoscopy (**d**). An esophageal stricture that developed after 3 esophageal stents (*black arrow*, **e**) that required serial dilations

A 71-year-old male with cuT2N2 distal esophageal adenocarcinoma in the presence of Barrett’s esophagus extending up to the thoracic inlet underwent 3HMIE after neoadjuvant chemoradiation. A gastric conduit was constructed via serial firing of staplers, and its perfusion was confirmed with Pinpoint fluoroscopy (Fig. 2a). Once again, anastomosis was created in the left neck using staplers below the transition line. On POD 5, bilious chest tube drainage was noted. Upon endoscopy, a 2 cm ischemic area was found just distal to the anastomosis. Therefore, a 23 mm × 155 mm pcSEMS was placed with its proximal end at the upper esophageal sphincter (Fig. 2b) and bridled with umbilical tape. His neck incision was drained. Despite the stent, the patient had persistent bile leak into the chest tube. It was felt that perhaps given the site of the leak in the thoracic inlet, it was not appropriately covered by the pcSEMS. Thus, he returned to the operating room for thoracoscopic decortication of the right lung. Upon stent removal, a 5 cm long dehiscence in the conduit was visualized along the gastric staple line (Fig. 2c). A V-Loc (Covidien, Mansfield, MA) 2-0 prolene suture was used thoracoscopically to reapproximate the gastric staple line dehiscence. Despite initial resolution of the bile leak, bilious chest tube output recurred in the next 48 h. On repeat endoscopy, the dehisced area was seen with inadequate closure (Fig. 2d). Since a pcSEMS failed the first time, a fully-covered 100 mm × 23 mm SEMS was placed. Eighteen days later, repeat endoscopy and stent removal showed a well-healing gastric conduit with a small, contained area of leakage. This time, a 23 mm × 155 mm pcSEMS was inserted to exclude the area of leakage and prevent stent migration. Three weeks later, the third stent was removed, and the gastric conduit was completely healed. His final pathology was ypT1aN0 stage IA esophageal adenocarcinoma. This patient also developed a stricture (Fig. 2e) that required serial balloon dilations. However, he has overall done well and is asymptomatic 9 months after his original procedure.

## Discussion

In the setting of 3HMIE with primary anastomosis at the cervical region, placement of esophageal stents has been anecdotally viewed as intolerable and technically difficult. Here, we report two patients with gastric conduit dehiscence after 3HMIE who were successfully managed endoscopically with temporary SEMS placement with concomitant chest washout that allowed gastric conduit salvage. Although both patients developed a post-operative stricture that required dilations, we believe that they were related to the original dehiscence and not the stent themselves.

Careful postoperative monitoring of all esophagectomy patients with high suspicion for anastomotic leakage is crucial in optimizing postoperative outcome. Typically, patients with conduit loss have an initial insidious course prior to clinical deterioration, and early identification of conduit ischemia is critical in decreasing the morbidity and mortality. Both of our patients underwent prompt evaluation for anastomotic leak prior to clinical deterioration and were found to have conduit dehiscence. Of note, neither of these two patients required vasopressors intra-operatively or post-operatively that could have resulted in conduit compromise and neither of them had any evidence of conduit malperfusion/ significant necrosis.

In review of the literature, there is one previous report of successful management of cervical esophago-gastric conduit disruption with SEMS [2]. However, to the best of our knowledge, using SEMS in the setting of conduit dehiscence and contamination of the thoracic cavity after 3HMIE with high anastomosis has never been reported.

## Conclusions

We therefore recommend the use of endoscopic stents in selected cases of gastric conduit dehiscence after 3HMIE in an effort to salvage the conduit, based on the clinical status of the patient, expertise of the surgeon, and experience of intensive care units that can manage such critically-ill patients. Albeit, we are not suggesting that if significant conduit necrosis is found or ischemia is suspected, that a stent be utilized as a salvage strategy; as those patients would need to have their conduit taken down with delayed reconstruction.

## Abbreviations

3HMIE: Three-hole minimally invasive esophagectomy
pcSEMS: Partially-covered self-expanding metal stent
SEMS: Self-expanding metal stent

## References

[1] Dickinson KJ, Blackmon SH (2015). Management of conduit necrosis following esophagectomy. Thorac Surg Clin.

[2] Oshikiri T, Yamamoto Y, Miki I (2015). Conservative reconstruction using stents as salvage therapy for disruption of esophago-gastric anastomosis. World J Gastroenterol.

